# Editorial: Roles of Fc Receptors in Disease and Therapy

**DOI:** 10.3389/fimmu.2020.01232

**Published:** 2020-06-12

**Authors:** Latha P. Ganesan, Mark S. Cragg, Gestur Vidarsson

**Affiliations:** ^1^Department of Internal Medicine, The Ohio State University, Columbus, OH, United States; ^2^Antibody and Vaccine Group, Centre for Cancer Immunology, Cancer Sciences, Faculty of Medicine, University of Southampton, Southampton, United Kingdom; ^3^Sanquin Research and Landsteiner Laboratory, Department of Experimental Immunohematology, Amsterdam UMC, University of Amsterdam, Amsterdam, Netherlands

**Keywords:** antibody, Fc gamma receptor (FcγR), immune response, disease, therapy

The humoral immune response is one of the central tenets of mammalian immunity. Delivered through the production of antibodies of multiple classes (IgG, IgM, IgA, IgD, and IgE) and sub-classes (e.g., IgG1, IgG2, IgG3, and IgG4 in humans) their activities are achieved through their inherent ability to bind with exquisite specificity to a given target antigen and then engage various immune effector functions to elicit the appropriate response. Chief amongst these are cellular immune effectors such as macrophages, NK cells, and neutrophils which are engaged through their expression of Fc receptors (FcR), binding the Fc portion of the immunoglobulins. Accordingly, different classes and isotypes of antibody engage a selection of different FcR. For example in the murine system there are receptors that are specific for IgG, IgM, IgE as well as receptors that are dually-specific for IgM and IgA with paralogues in human cells. A bewildering array of immune and non-immune cells express these various receptors in different combinations, leading to a highly complex system for regulating and evoking antibody responses. Various FcR evoke cellular activation (FcγRIIa and FcγRIIa), whereas others are inhibitory (FcγRIIb), with still others being capable of evoking intracellular transport and recycling of IgG (FcRn) to establish long serum half-lives. Clearly, careful regulation of expression, signaling and modulation is required for a healthy, well-functioning and balanced immune system. In this Research Topic, a series of articles are provided to reveal comprehensive insights on the role of these various FcR in health and disease, taking into account the wide spectrum of receptors and cells expressing them. Most importantly the insights presented in these articles pave the way for powerful immunotherapies and emerging principles about how FcR can be exploited for therapeutic purposes for various diseases, including infectious diseases, autoimmune diseases, and cancer.

In total, 6 original research articles were contributed on the various topics, spanning the genetics and function of the disparate FcγR. While Kerntke et al. revisited the question of the number and expression pattern of FcγR on myeloid cells, Nagelkerke et al. dissected the genetic variation within the family, including duplications and deletions within the low affinity FcγR-locus. How the GPI-linked FcγRIIIb affects tumor cell killing by PMN through therapeutic monoclonal antibodies is furthermore tackled by Treffers et al. while Kang et al. describes a new re-engineered IgG molecule that selectively engages FcγRIIIa-V158 for enhanced therapeutic benefit through a single FcγR. Brandsma et al. also investigated the differential capacity of tumor killing through FcR that engage different antibody isotypes, specifically addressing the role of FcαR vs. FcγR. Parameters affecting the function of FcRn were also tackled. Finally, Kendrick et al. mathematically modeled FcRn kinetics and suggest a novel reduced-order model based on a new expression for the fractional catabolic rate that can be used to predict plasma IgG responses.

This Research Topic also features 18 Review Articles spanning these disparate areas. FcRn is tackled by Pyzik et al. and Nagelkerke et al. also contributes a comprehensive review of FcγRII-FcγRIII genetics. Anania et al. systematically discuss the structure-function relationship of FcγRII receptors, while the contribution of FcγRIIb in the development of autoimmune diseases in mouse models gets a comprehensive assessment by Verbeek et al.. Breedveld and van Egmond review pathologies and new opportunities resulting from targeting FcαR. In addition, Foss et al. extend the scope of this topic to the cytosolic FcR, TRIM21, while Liu et al. and Kubagawa et al. discuss the role of the IgM binding, FcμR in immunity. The role of FcR in infectious diseases and vaccine development is covered by Boudreau and Alter, discussing FcR and their role in the protection against influenza infection and future prospects to leverage FcR immune activity for the development of vaccines with Jenks et al. focusing on the subversion of immune responses by FcR encoded by Herpes simplex virus. The involvement of FcR in various inflammatory diseases such as rheumatoid arthritis, systemic lupus erythematosus, and immune thrombocytopenia with a focus on antibody-mediated autoimmunity is covered by Mkaddem et al.. This includes the mechanism of FcR-receptor-mediated inflammation and how to potentially exploit this knowledge therapeutically. Katsinelos et al. focuses on the role of antibodies and receptors involved in neurodegeneration during Alzheimer's and Parkinson's disease, while Castro-Dopico and Clatworthy discuss the role of FcR in inflammatory diseases of the gut, namely inflammatory bowel diseases. Patel et al. discusses the multiple variables that are at play in the interface between target and effector cells through IgG-FcγR engagement, with a focus on the largely undescribed role for FcγR-glycosylation in mediating the underlying recognition events. FcR signaling is also specifically covered by Gomez et al. for FcεRI in Allergic disease, including seasonal rhinitis, atopic dermatitis, urticaria, anaphylaxis, and asthma, while Koenderman et al. reviews how the activation status of FcR can be affected by inside-out signaling. Finally, the importance of FcR in cancer and cancer therapies, in particular, the role of checkpoint inhibitors therein, is given comprehensive review by Chen et al. and special focus on FcγRIIb mediated antitumor immunity by Teige et al.

Overall, it is clear that the knowledge acquired from the articles contained within this special issue highlights the complexity of the FcR family and their importance in multiple aspects of health and disease. However, equally clear is the fact that this family of receptors, despite being investigated for over 4 decades, still harbors many secrets, reinforcing that we still lack a complete understanding of their complex regulation, interaction and impacts. As central to humoral immunity, modulating disease pathogenesis and acting as a key determinant of antibody therapeutics, it is also similarly evident that further research in this area is still warranted. We look forward to seeing what the intensive study of these receptors shows in the coming decade.

## Author Contributions

All authors listed have made a substantial, direct and intellectual contribution to the work, and approved it for publication.

## Conflict of Interest

The authors declare that the research was conducted in the absence of any commercial or financial relationships that could be construed as a potential conflict of interest.

